# Systems Biology-Based Investigation of Cellular Antiviral Drug Targets Identified by Gene-Trap Insertional Mutagenesis

**DOI:** 10.1371/journal.pcbi.1005074

**Published:** 2016-09-15

**Authors:** Feixiong Cheng, James L. Murray, Junfei Zhao, Jinsong Sheng, Zhongming Zhao, Donald H. Rubin

**Affiliations:** 1 State Key Laboratory of Biotherapy/Collaborative Innovation Center for Biotherapy, West China Hospital, West China Medical School, Sichuan University, Chengdu 610041, Sichuan, China; 2 Department of Biomedical Informatics, Vanderbilt University School of Medicine, Nashville, Tennessee, United States of America; 3 Zirus, Incorporated, Buford, Georgia, United States of America; 4 Center for Precision Health, School of Biomedical Informatics, The University of Texas Health Science Center at Houston, Houston, Texas, United States of America; 5 Division of Infectious Disease, Department of Medicine, Vanderbilt University School of Medicine, Nashville, Tennessee, United States of America; 6 Department of Psychiatry, Vanderbilt University School of Medicine, Nashville, Tennessee, United States of America; 7 Department of Cancer Biology, Vanderbilt University School of Medicine, Nashville, Tennessee, United States of America; 8 Department of Pathology, Microbiology and Immunology, Vanderbilt University School of Medicine, Nashville, Tennessee, United States of America; 9 Research Medicine, Veterans Tennessee Valley Healthcare System, Nashville, Tennessee, United States of America; National University of Singapore, SINGAPORE

## Abstract

Viruses require host cellular factors for successful replication. A comprehensive systems-level investigation of the virus-host interactome is critical for understanding the roles of host factors with the end goal of discovering new druggable antiviral targets. Gene-trap insertional mutagenesis is a high-throughput forward genetics approach to randomly disrupt (trap) host genes and discover host genes that are essential for viral replication, but not for host cell survival. In this study, we used libraries of randomly mutagenized cells to discover cellular genes that are essential for the replication of 10 distinct cytotoxic mammalian viruses, 1 gram-negative bacterium, and 5 toxins. We herein reported 712 candidate cellular genes, characterizing distinct topological network and evolutionary signatures, and occupying central hubs in the human interactome. Cell cycle phase-specific network analysis showed that host cell cycle programs played critical roles during viral replication (e.g. *MYC* and *TAF4* regulating G0/1 phase). Moreover, the viral perturbation of host cellular networks reflected disease etiology in that host genes (e.g. *CTCF*, *RHOA*, and *CDKN1B*) identified were frequently essential and significantly associated with Mendelian and orphan diseases, or somatic mutations in cancer. Computational drug repositioning framework via incorporating drug-gene signatures from the Connectivity Map into the virus-host interactome identified 110 putative druggable antiviral targets and prioritized several existing drugs (e.g. ajmaline) that may be potential for antiviral indication (e.g. anti-Ebola). In summary, this work provides a powerful methodology with a tight integration of gene-trap insertional mutagenesis testing and systems biology to identify new antiviral targets and drugs for the development of broadly acting and targeted clinical antiviral therapeutics.

## Author Summary

Infectious diseases result in millions of deaths and cost billions of dollars annually. Hence, there is urgency for developing more innovative and effective antiviral therapeutics. In this study, we used libraries of randomly mutagenized cells to discover cellular genes that are essential for the replication of 10 distinct cytotoxic mammalian viruses. We herein reported over 700 candidate cellular genes, over 20% of which were independently selected by multiple viruses in one or more cell types. Using systems biology-based analysis, we found that host genes associated with viral replication tended to occupy central hubs in the human protein interactome and to be ancient genes with low evolutionary rates, compared to non-virus-associated genes. Cell cycle phase-specific sub-network analysis showed that host cell cycle program played important roles during viral replication by regulating specific cell cycle phases. Moreover, we presented novel evidences to suggest that host genes supporting viral replication were frequently implicated in Mendelian and orphan diseases, or played critical roles in cancer. Importantly, we found approximately 110 new putative druggable antiviral targets by merging genome-wide gene-trap insertional mutagenesis, drug-gene network, and bioinformatics data. Furthermore, we have demonstrated the use of a computable representation of genetic testing to effectively identify new potential antiviral indications for existing drugs. In summary, this study presents new and important methodologies for developing broadly active antiviral therapeutics.

## Introduction

Infectious diseases result in millions of deaths and cost billions of dollars annually [1]. As of 2012, 35.3 million people worldwide were living with human immunodeficiency virus (HIV), and an estimated 1.6 million acquired immunodeficiency syndrome (AIDS)-related deaths were reported in 2012 [2]. In March 2014, the Worth Health Organization reported a major Ebola virus outbreak in the western African nation of Guinea. As of March 25, 2015, over 26,000 suspected Ebola-infected cases had been identified, with over 10,000 deaths, and these numbers may be vastly underestimated [3]. Infections by the Ebola and Marburg filoviruses cause a rapidly fatal hemorrhagic fever in humans for which no approved antiviral agents are available [4–6]. Traditional antiviral drug discovery pipelines have yielded notable successes in recent years. However, two factors continue to provide commercial and medical incentives for developing more innovative and effective antiviral therapeutics, namely the propensity of viruses to develop drug resistance and the side effects caused by antiviral agents [7,8]. With faster development times, increased safety, and decreased pharmacokinetic uncertainty, the prospect of drug repositioning (finding new indications for existing FDA-approved drugs) is emerging as a promising alternative to traditional drug design and offers an improved risk-benefit trade-off in combating infectious diseases [9–11].

Viruses require host cellular factors for successful replication. A comprehensive systems-level investigation of the virus-host interactome is crucial for understanding the roles of host factors with the end goal of discovering new druggable antiviral targets [8,12]. In this regard, quantitative temporal viromics [13] and viral open reading frames [14] can be useful in studying the virus-host interactome [12–15]. However, the incorrect assignment of biological activities to viral and host factors, and the limited scale of experimental techniques have limited these approaches [8,16]. Gene-trap insertional mutagenesis is a promising approach for elucidating host cellular network perturbations during viral replication [17,18]. Gene-trap insertional mutagenesis is a high-throughput forward genetics approach to randomly disrupt (trap) host genes and discover cellular genes that are essential for viral replication, but not for host cell survival [19]. This approach is based on two important principles: (i) viral infection must be toxic to the chosen host cell line, and (ii) disrupting a gene critical for completing the viral life cycle confers survivability during subsequent viral selection, provided that the host cell can survive following reduced or abolished expression of the mutagenized gene.

**Fig 1:**
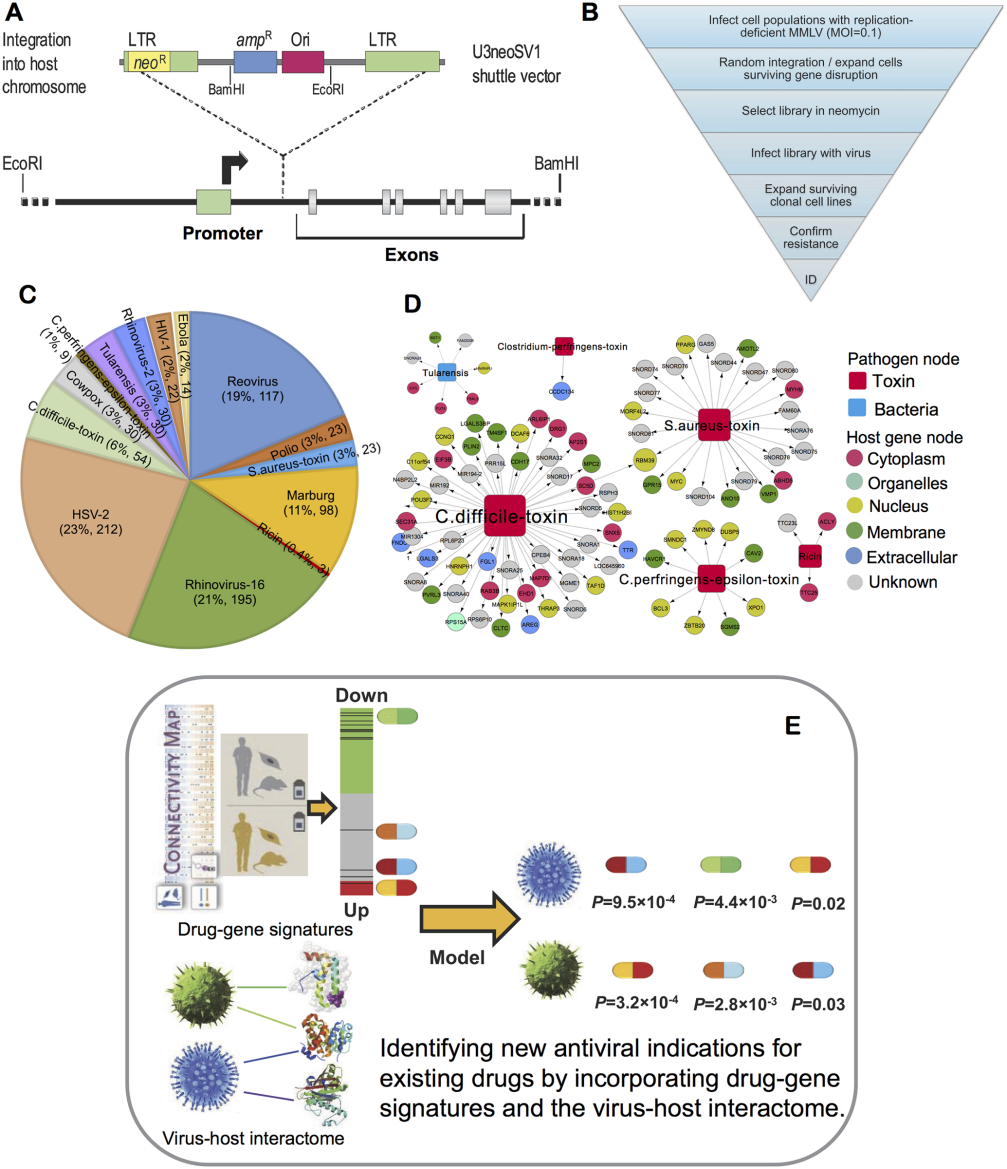
Diagram of the integrative antiviral drug discovery pipeline. (**A**) The gene-trap insertional mutagenesis approach employs an MMLV-based shuttle vector that randomly integrates into host cell chromosomes and contains a promoterless neomycin-resistance gene. Shuttle vector integration between a host-cell promoter and an early exon disrupts (traps) the gene, allowing neomycin selection and derivation of a gene-trap library. (**B**) Host genes mediating the toxic effects of lytic viral replication or exposure to toxins were identified by: (i) selecting gene-trap libraries in neomycin; (ii) exposing gene-trap library cells to a lytic virus or a toxin; (iii) isolating surviving clones; (iv) resistance confirmation in surviving clones following exposure to a 10-fold higher dose of the virus or toxin studied; and (v) identification of the trapped gene by digesting genomic DNA to liberate shuttle vectors, self-ligation, bacterial transform, ampicillin selection, and sequencing trapped genes in the recovered plasmids. (**C**) Distribution of newly discovered virus-host interaction pairs for 10 viruses, 1 bacterium, and 5 toxins. (**D**) Global pathogen-host interaction network identified by genome-wide gene-trap insertional mutagenesis, where toxins and bacteria are represented by red and cyan squares respectively. The host cell gene products (circles) are colored based on their subcellular locations collected from the LocDB (https://www.rostlab.org/services/locDB/). (**E**) Identification of candidates for antiviral drug repositioning approach by incorporating drug-gene signatures from the Connectivity Map into the global virus-host interactome.

In this study, we incorporated gene-trap insertional mutagenesis, known drug-gene signatures, and bioinformatics analysis into an integrated antiviral drug discovery pipeline (Fig 1). We used libraries of randomly mutagenized cells to discover host cellular genes that are essential for the replication of 10 distinct cytotoxic mammalian viruses, 1 gram-negative bacterium, and 5 bacterial toxins. We present novel evidence to suggest that host genes supporting viral infection are frequently implicated in Mendelian and orphan diseases, or play roles in cancer. Furthermore, we identified antiviral targets that are likely to be inhibited by known drugs, allowing us to predict several new antiviral indications (i.e., anti-Ebola) for existing drugs. In summary, we present a powerful approach for identifying potential druggable targets and existing drugs with good pharmacokinetics profiles for the development of broadly active antiviral therapeutics.

## Results

### Expanding the known virus-host interactome by gene-trap insertional mutagenesis

An integrated antiviral drug-discovery pipeline was developed that involves gene-trap insertional mutagenesis, consolidated drug-gene signatures, and bioinformatics analysis to rank candidate antiviral targets and identify potential antiviral indications for existing drugs (Fig 1). Specifically, we used genome-wide gene-trap insertional mutagenesis to identify new virus-host interactions by the following 6 steps: (i) random integration of an insertional mutagen shuttle vector containing a promoterless neomycin-resistance gene; (ii) neomycin selection of cells expressing neomycin aminotransferase; (iii) cytotoxic viral infection; (iv) resistance confirmation by re-infecting surviving clones at a 10-fold higher multiplicity of infection (MOI); (v) shuttle vector recovery from resistant clones (genomic DNA digestion, self-ligation, bacterial transformation, and ampicillin selection); and (vi) sequencing of trapped genes from bacterial colonies (Fig 1A and 1B). In this manner, we identified approximate 700 candidate host genes (Fig 1C and S1 Table) mediating the cytotoxic effects of 10 viruses (cowpox virus, Ebola virus, HIV-1, *Herpes simplex* viruses (HSV)-1 and HSV-2, Marburg virus, poliovirus (Polio), reovirus, and rhinovirus-2 and -16), of which 20% were identified in studies with multiple viruses in one or more cell types. Following the same general method for gene-trap studies outlined above, we also identified 97 host genes (Fig 1D and S1 Table) mediating the lytic effects of *Francisella tularensis* (*tularensis*) and 5 toxins (*Clostridium difficile* TcdB toxin, *Clostridium perfringens* ε toxin, *Helicobacter pylori* vacuolating toxin, *Staphylococcus aureus* α toxin, and ricin toxin). Encouraged by these findings, we then developed a systems biology-based pipeline to characterize the candidate cellular antiviral targets through network approaches and bioinformatics analysis. Finally, we computationally predicted several new antiviral indications for existing drugs by incorporating drug-gene signatures from the Connectivity Map (CMap) [20] into the global virus-host interactome (Fig 1E).

**Fig 2:**
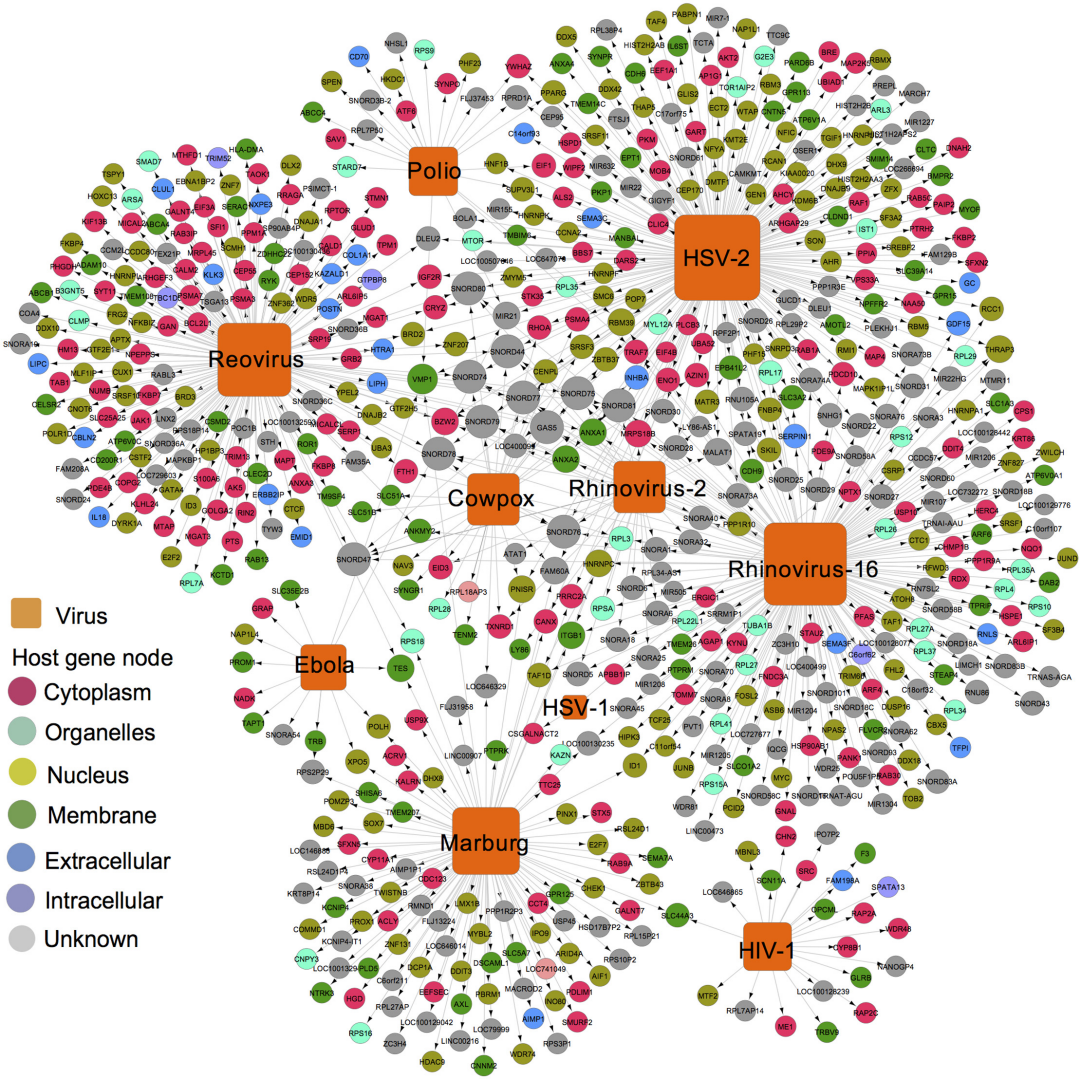
The newly identified virus-host interaction networks by gene-trap insertional mutagenesis. The nodes (squares) are viruses, host cell gene products (circles) are colored based on their subcellular locations collected from LocDB (https://www.rostlab.org/services/locDB/), and edges (lines) denote interactions identified by gene-trap insertional mutagenesis.

We next plotted the 900 newly discovered pathogen-host interactions newly discovered in this study using two bipartite graphs: a toxin-host interaction network (Fig 1D) and a virus-host interaction network (Fig 2), where nodes represent 712 host genes (circles), 10 viruses (orange squares), 1 gram-negative bacterium (cyan square), and 5 toxins (red squares), and where edges represent interactions identified by gene-trap insertional mutagenesis. The host genes are grouped based on human protein subcellular locations, such as membranes, the cytoplasm, organelles, and the nucleus collected from the LocDB [21]. A detailed list of these data is provided in S1 Table. Identifying toxin-host interactions may provide important targets for preventing toxin-induced cytotoxicity in toxin-producing bacteria. Recently, our group identified poliovirus receptor-like 3 (PVRL3) as a cellular factor necessary for *Clostridium difficile* TcdB-induced cytotoxicity, using gene-trap insertional mutagenesis [22]. Thus, the toxin-host interactions identified by gene-trap insertional mutagenesis shown in Fig 1D could provide useful resources for developing novel antibiotic therapies.

**Fig 3:**
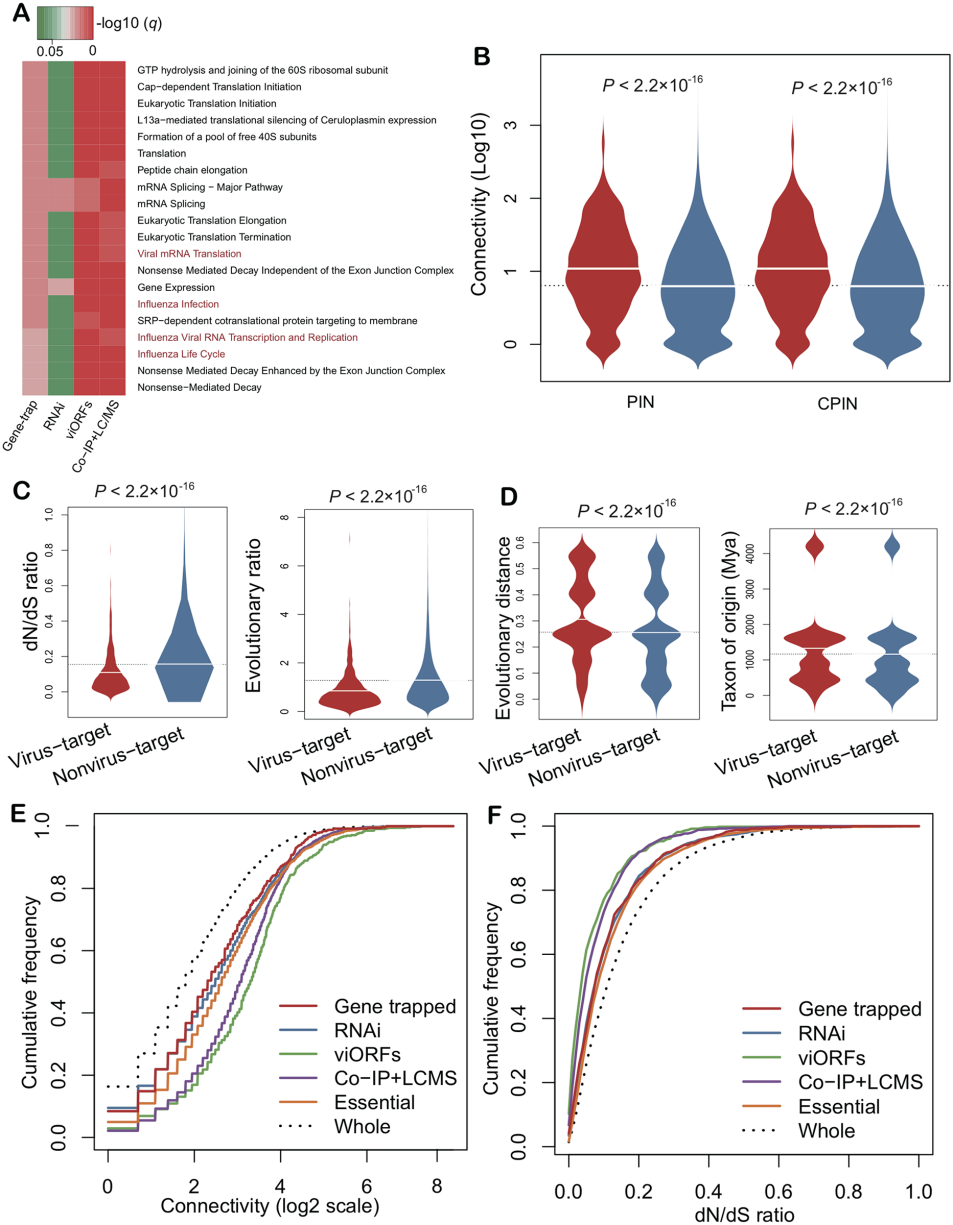
Bioinformatics analysis and network topological and evolutionary characteristics of host genes mediating viral replication. (**A**) Reactome pathway enrichment analysis of four different host cellular gene sets identified by gene-trap insertional mutagenesis (trapped genes), previous RNA interference (RNAi) screening studies, viral open reading frames (viORFs), and co-immunoprecipitation and liquid chromatography-mass spectrometry (Co-IP+LC/MS) (S1 Table). (**B**) Boxplots showing the connectivity distribution of virus host genes (red) versus non-virus-host genes (light blue) in the physical protein interaction network (PIN) and large-scale computationally predicted protein interaction network (CPIN). (**C**) and (**D**) Evolutionary characteristics of virus-host genes (red) versus non-virus-host genes (light blue). (**E**) Node connectivity distribution of host genes identified by gene-trap insertional mutagenesis and three published gene sets and all proteins (Whole) in PIN. (**F**) Gene *dN/dS* ratio cumulative distribution for four different gene sets and whole human genome (Whole). Mya: million years ago. *P* values in **B-D** were calculated via Wilcoxon rank-sum test.

To further evaluate the quality of host genes identified by gene-trap insertional mutagenesis, we compared our network to three previously independent networks. In total, we assembled 2,855 known virus-host interactions connecting 2,443 host genes and 55 pathogens identified from RNA interference (RNAi), 579 host proteins mediating 70 innate immune-modulating viral open reading frames (viORFs) [14], and 1,292 host genes mediating influenza-host interactions identified by co-immunoprecipitation and liquid chromatography-mass spectrometry (Co-IP+LC/MS) [23], respectively (S1 Table). Several critical viral replication-related pathways, such as viral mRNA translation, influenza viral RNA transcription and replication, influenza infection, and influenza life cycle were significantly (adjusted p-value [q] < 0.01) enriched among the host genes identified in our gene-trap insertional mutagenesis, viROFs, and Co-IP+LC/MS studies, but not in the RNAi gene set (Fig 3A, S2 and S3 Tables).

### Network centrality of pathogen-target genes in the human protein interaction network

Of 712 host genes identified by gene-trap insertional mutagenesis, there was enrichment for genes associated with innate immunity (*P* = 4.7 × 10^−3^, Fisher’s exact test, S1A Fig), suggesting that the identified host gene set may mediate immune responses [14]. Essential genes, whose knockout result in lethality or infertility, are important for studying the robustness of a biological system [24]. Furthermore, there was also a significant enrichment for essential genes (*P* = 1.0 × 10^−5^, S1B Fig).

To further investigate the biological functions of the identified virus-target genes, we further examined topological network features for virus-target gene products (proteins) in the human protein interactome. Considering that the current publicly-available human protein interaction databases are still incomplete, we constructed 5 different, yet complementary human protein interaction networks: a global physical protein interaction network (PIN), an atomic resolution three-dimensional structural protein interaction network (3DPIN), a kinase-substrate interaction network (KSIN), an innate immunity protein interaction network (INPIN), and a broad context computationally predicted protein interaction network (CPIN), based on two previous studies [25,26]. Fig 3B shows that the connectivity of virus-target proteins was significantly stronger than non-virus target proteins in PIN (*P* < 2.2 × 10^−16^, Wilcoxon test) and CPIN (*P* < 2.2 × 10^−16^), respectively. In addition, we defined “hubs” as those nodes that ranked in the top 20% of the connectivity distribution, as done previously [25,26]. We found that virus-target proteins were significantly enriched in hubs in all 5 human protein interaction networks (S4 Table). Moreover, the virus-target proteins showed a tendency for greater enrichment of hubs in innate immunity PIN [27] than in CPIN (*P* < 0.01), suggesting that the immune system plays an important role during viral replication, consistent with the enrichment for cellular genes associated with innate immunity shown in S1A Fig

In addition, we investigated the connectivity distribution of our 712 host genes with three published host gene sets. We found a comparable connectivity distribution of our 712 host genes with the RNAi gene set, although they were marginally lower in terms of significance than that observed with the viORFs and Co-PI+LC/MS gene sets (Fig 3E). These observations suggest the reliability of gene-trap insertional mutagenesis, relative to results obtained using other technologies such as RNAi, Co-IP+LC/MS, and viORFs.

### Purifying selection and evolutionary origins of virus-target genes

To provide insight into the evolutionary factors underlying the selection of host genes used by viruses, we examined the selective pressure and evolutionary rates of the virus-target genes identified. We computed non-synonymous and synonymous substitution rate ratios (*dN/dS* ratios) using human-mouse orthologous gene pairs (see Methods). A *dN/dS* ratio of 1 signifies neutral evolution, a ratio of < 1 indicates purifying selection, and a ratio of > 1 indicates positive Darwinian selection. The boxplots in Fig 3D show that virus-target genes tend to undergo purifying selection (i.e., the selective removal of alleles that are deleterious) in human protein evolutionary histories. Moreover, virus-target genes displayed stronger purifying selection (lower *dN/dS* ratios and evolutionary rate ratios) than did non-virus target genes (*P* < 2.2 × 10^−16^, Wilcoxon rank-sum test), as shown in Fig 3D. For example, several genes with the lowest *dN/dS* ratios (0) such as *RAB1A* [28], *PCBP1* [29], *PCBP2* [30], and *ARF6* [31] were previously reported to be involved in viral replication or antiviral signaling pathways. However, only one gene (*DEFB118*), which was also implicated in viral replication-related pathway [32] had a *dN/dS* ratio large than 1 (1.1).

The evolutionary history of a protein sequence often reflects its functional evolution. We next investigated the evolutionary origin of virus-target gene products. The average time of divergence (1348.6 ± 20.0 million years ago [Mya]) for virus-target gene products was significantly longer than that of non-virus target gene products (1131.3 ± 8.7 Mya, *P* < 2.2 × 10^−16^; Fig 3D). Furthermore, the average evolutionary distance of virus-target gene products was also significantly higher than that observed for non-virus target gene products (*P* < 2.2 × 10^−16^; Fig 3D). We next compared the *dN/dS* ratio distribution for our 712 host genes with that of three published host gene sets. Compared with our set of 712 host genes, a similar trend was observed with the RNAi gene set (Fig 3F).

### Regulating the host cell cycle program

**Fig 4:**
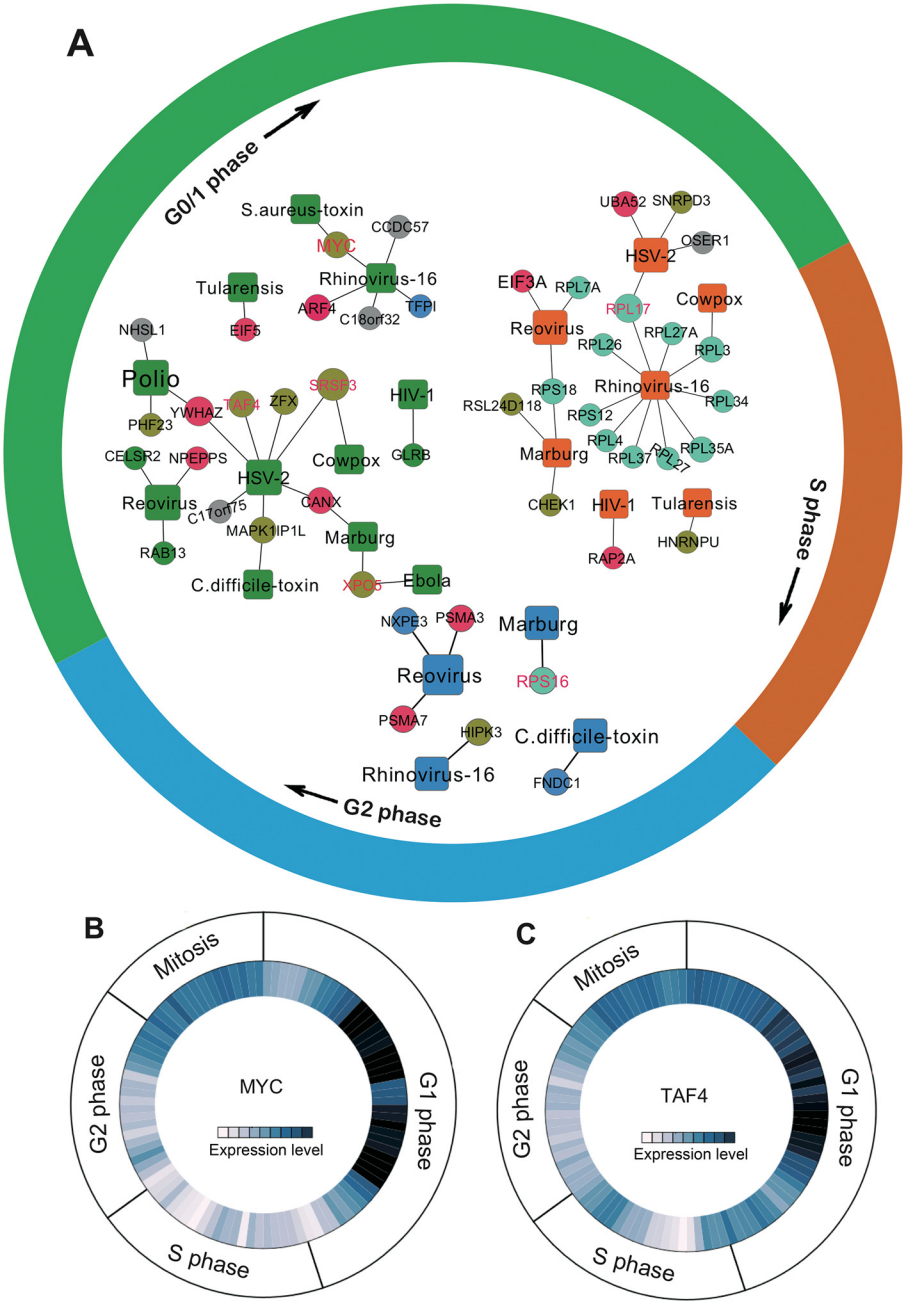
Human cell cycle phase-specific virus-host gene network. (**A**) Human cell cycle phase-specific virus-host gene network for host genes identify by gene-trap insertional mutagenesis. The concise overview of cell cycle regulation for gene *MYC* (**B**) and *TAF4* (**C**). Dark color represents high expression across different cell cycle phases. Images in **B** and **C** are prepared by Cyclebase 3.0 (http://www.cyclebase.org).

Most viruses are known to regulate host cell cycle program [33,34]. We assembled 986 human host cell cycle genes mediating G0/1, S, and G2 phases from a previous study [35]. We found that the 712 host genes identified by gene-trap were significantly enriched in terms of human cell cycle genes (*P* = 6.5 × 10^−5^, Fisher’s exact test). We next built a cell cycle phase-specific sub-network to examine the cell cycle programing mechanism for our host gene set. Fig 4A shows that several genes important for viral replication also mediate progression though G0/G1 phase, including *MYC*, *ARF4*, *SRSF3*, *TAF4*, *XPO5*, and *EIF5*. *ARF4* promotes enterovirus 71 replication [36], susceptibility to *Chlamydia trachomatis* and *Shigella flexneri* [37], and dengue flavivirus secretion [38]. Two previous studies have suggested that *TAF4* plays critical roles in herpes simplex virus type 1 infection [39] and transcriptional activation of Epstein-Barr virus [40]. Here, we identified that *TAF4* might mediate HSV-2 replication by regulating G0/1 phase identified via gene-trap. Furthermore, cell cycle-specific expression analysis using Cyclobase [41] further confirmed that *TAF4* regulates cell cycle in G1 phase in Fig 4C. *MYC*, encoding c-Myc, is involved in the replication of multiple viruses, such as Epstein-Barr virus [42,43]. Here, we found that *MYC* may mediate rhinovirus-16 replication by regulating G0/G1 phase (Fig 4A and 4B). In addition to G0/1 phase, we also found that several genes (e.g. *RPL17* and *RPS16*) regulate S or G2 phase transition, in addition to viral replication (Fig 4A). *RPL17*, encoding 60S ribosomal protein L17 important for protein synthesis, plays critical roles in the replication of several viruses [44], such as hepatitis C virus and HSV-1. Collectively, these observations further suggested that the host cell cycle program plays important roles during viral replication by regulating specific cell cycle phases.

### Viral perturbations of cellular networks reflect disease etiology

**Fig 5:**
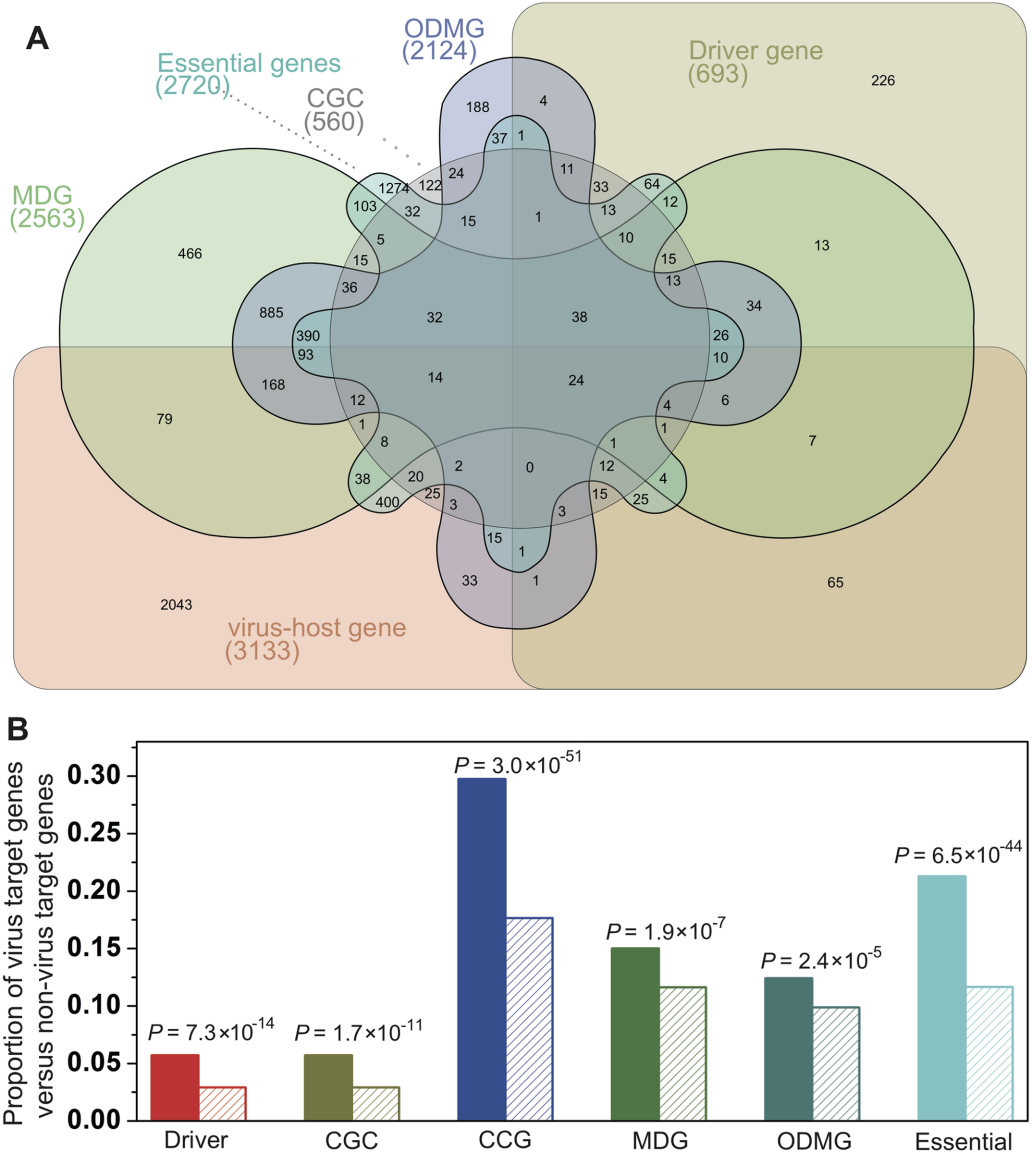
Disease etiology analysis of virus target genes. (**A**) Venn diagram denoting the overlap among virus-target genes (Host genes), genes whose mutations are significantly associated with cancer (Driver), genes in the Cancer Gene Census (CGC, experimentally validated cancer genes), the catalogue of cancer genes (CCG), Mendelian disease genes (MDG), orphan-disease mutated genes (ODMG), and essential genes (Essential). (**B**) Disease gene enrichment analysis of virus-target genes (solid bars) versus nonvirus-target genes (striped bars). *P* values are calculated using Fisher’s exact test.

Understanding the interrelations between cellular host genes targeted by viral proteins and disease-susceptibility genes may reveal critical information for disease etiology [45,46]. We investigated the overlap between virus-target genes and the gene sets implicated in Mendelian diseases, orphan diseases, and cancer (Fig 5A). Fig 5B shows that virus-target genes are significantly enriched in Mendelian disease genes (MDG; *P* = 1.9 × 10^−7^), orphan disease-mutated genes (ODMG; *P* = 2.4 × 10^−5^), and those in the catalogue of cancer genes (CCG *P* = 3.0 × 10^−51^).

**Fig 6:**
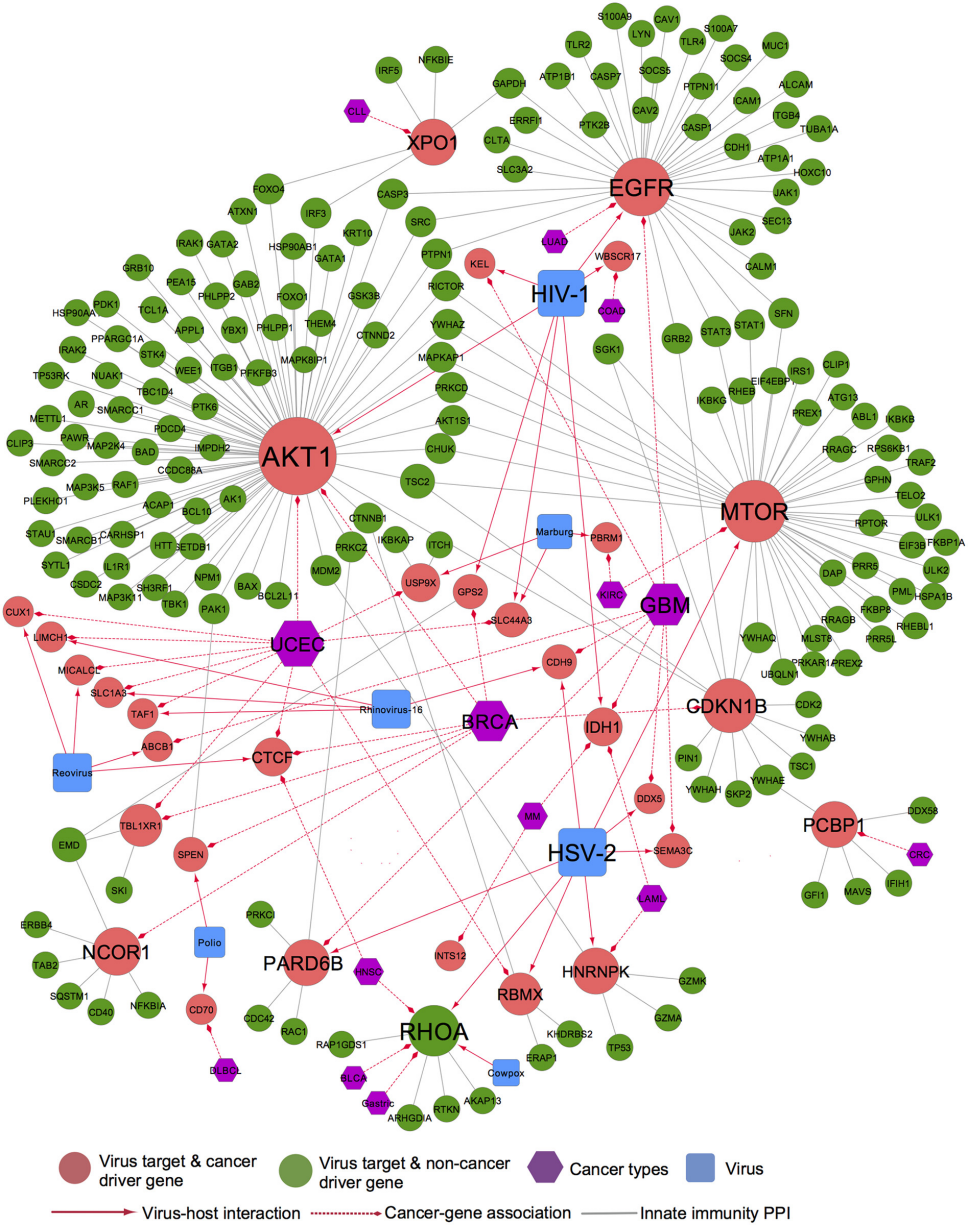
Novel viral perturbations of the innate immunity network reveal new cancer etiologies. In this network, nodes represent viruses (squares), cancer types (hexagons), and genes (circles). Edges represent virus-host interactions (solid red arrows), cancer-gene associations (striped red arrows), and innate immunity protein-protein interactions (solid gray lines). Various cancer types represented are abbreviated as follows: breast invasive carcinoma (BRCA), bladder urothelial carcinoma (BLCA), colon adenocarcinoma (COAD), diffuse large B-cell lymphoma (DLBCL), glioblastoma multiforme (GBM), head and neck squamous cell carcinoma (HNSC), acute myeloid leukemia (LAML), kidney renal clear cell carcinoma (KIRC), lung adenocarcinoma (LUAD), multiple myeloma (MM), and uterine corpus endometrial carcinoma (UCEC). Detailed data are provided in S5 Table.

A previous study has suggested that genomic variations and tumor viruses might cause cancer through related mechanisms [45]. Thus, we examined how virus-target genes promote tumorigenesis or are involved in cancer etiology. We compiled 384 genes that are significantly mutated in cancer (cancer-driver genes) from several large-scale cancer genome projects (S5 Table). Interestingly, a significant association (*P* = 7.3 × 10^−14^) was observed between the cancer-related genes and genes implicated in viral infection identified by our gene-trap studies and prior RNAi screens (Fig 5B). As shown in Fig 6 and S5 Table, 26 of the 384 cancer driver genes were identified in gene-trap studies with lytic viruses (such as *CTCF*, *RHOA*, *CDKN1B*, and *CUX1*), while 66 of the 384 genes were previously identified in RNAi screens (such as *PIK3CA*, *HRAS*, *EGFR*, *AKT1*, and *IDH1*). However, the overlap with the cancer gene set may be confounded by the facts that multiple species of cells were used in this study and that both immortalization and viral infection perturbed cellular pathways related to growth. This phenomenon was also discussed in a previous study [45].

The human *CTCF* gene encodes the CTCF transcriptional repressor by mediating transcriptional regulation, insulator activity, and the regulation of chromatin architecture [47]. Data from several recent cancer genome projects showed that *CTCF* mutations are significantly associated with breast cancer [48], head and neck cancer [49], and uterine cancer [50]. Interestingly, *CTCF* is involved in reovirus replication identified by gene-trap (Fig 6). Pre-clinical studies have suggested that treatment with reovirus is associated with significant anticancer activity in various cancer types, such as ovarian cancer [51] and colon cancer [51]. Furthermore, a recent study showed that infection with an oncolytic adenovirus (Ad315-E1A) or a replication-deficient recombinant adenovirus (Ad315-EGFP) significantly decreased cell viability and induced apoptosis of colon cancer cells *in vitro* and reduced tumor growth in a xenograft model by targeting *CTCF* binding sites (CCCTC) [52]. Thus, developing a novel reovirus that targets *CTCF* transcription factor binding sites by partial inhibition of viral replication or partial oncolytic activity may provide a potential strategy for targeted cancer therapy. Collectively, virus-host perturbation networks may shed valuable insight for prioritizing disease-associated or cancer-driver mutations [53].

### Identifying new antiviral targets and indications for existing drugs

**Fig 7:**
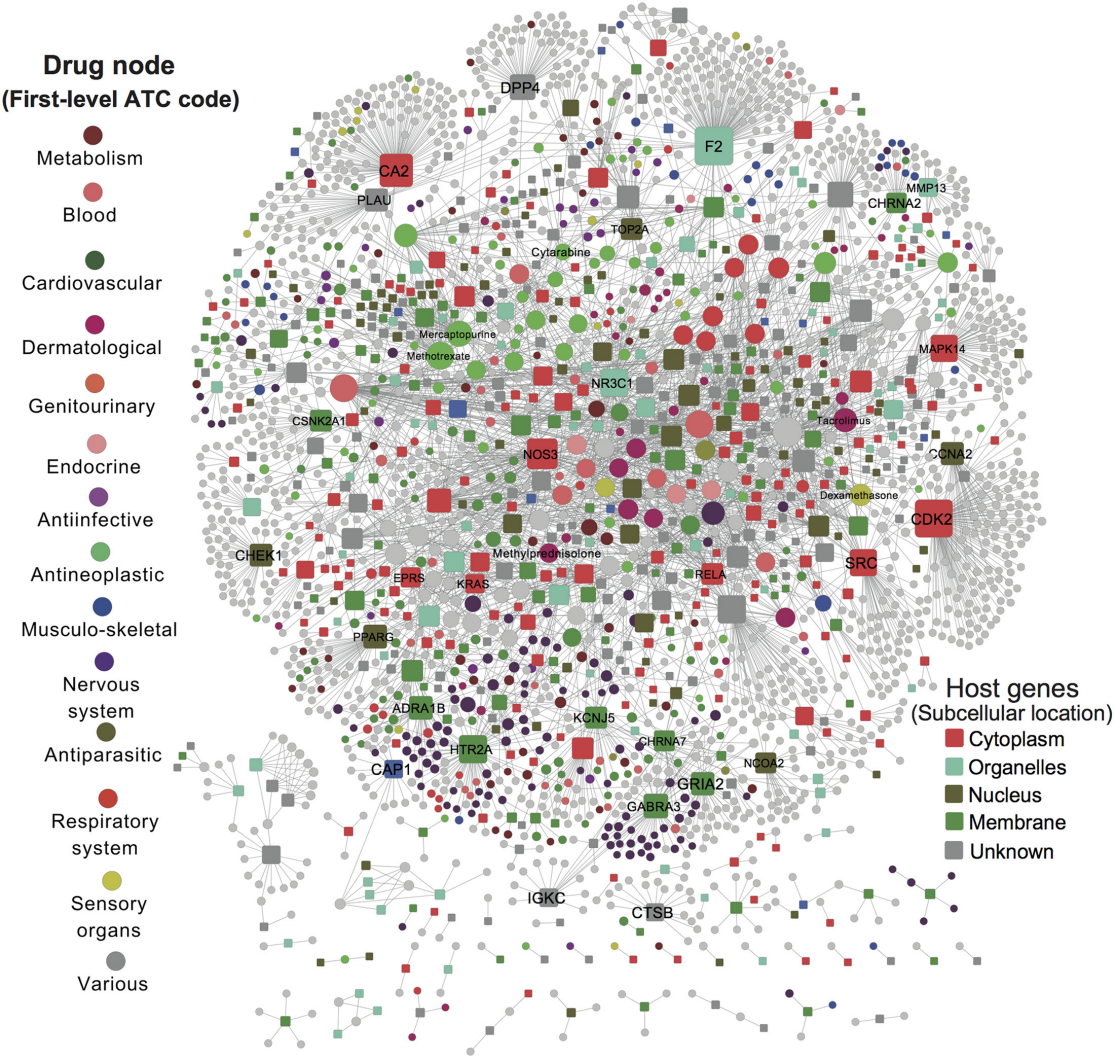
Global antiviral bipartite drug-target interaction network. In this network, nodes represent 691 virus-target genes (Host genes, squares) or known drugs (2,071) shown in circles, and where edges denote the interactions. Host gene products were colored based on their known subcellular locations. All drugs were grouped using the First-level anatomical therapeutic chemical (ATC) code classification system. Detailed data are provided in S7 Table.

To identify new druggable targets for antiviral pharmacotherapy, we cross-referenced all virus target genes (S1 Table) identified by previous global RNAi screens and gene-trap studies with 3 drug-target databases, namely DrugBank [54], Therapeutics Target Database [55], and PharmGKB [56]. In total, we found 615 virus target genes (110 host genes identified by gene-trap) whose products can be targeted by FDA approved drugs, investigational drugs, or pre-clinical agents, which are referred to here as “druggable virus-target genes.” We performed KEGG pathway analysis for these 615 druggable virus-target genes. The most significantly enriched pathways included Epstein-Barr virus infection (*q* = 7.0 × 10^−13^), osteoclast differentiation (*q* = 3.4 × 10^−7^), proteasome (*q* = 1.9 × 10^−7^), the neurotrophin signaling pathway (*q* = 1.1 × 10^−6^), ERBB signaling pathway (*q* = 2.0 × 10^−6^), influenza-A (*q* = 1.0 × 10^−5^), T cell receptor signaling pathways (*q* = 1.3 × 10^−5^), and the MAPK signaling pathway (*q* = 5.7 × 10^−5^, S6 Table). Fig 7 showed a bipartite drug-target interaction network connecting 691 virus-target genes (squares) and 2,071 existing drugs (circles). Multiple drugs exist for several gene products, including CDK2, NOS3, NR3C1, MAPK14, SRC, and CHEK1 (Fig 7 and S7 Table), providing new opportunities by targeting those genes for antiviral pharmacotherapy. Interestingly, most cancer drugs often target host genes mediating viral replication. KEGG pathway enrichment analysis showed that several of the most significant pathways are involved in cancer, such as chronic myeloid leukemia (*q* = 5.3 × 10^−10^), pathways in cancer (*q* = 1.9 × 10^−8^), prostate cancer (*q* = 2.3 × 10^−8^), and pancreatic cancer (*q* = 4.8 × 10^−8^). Fig 7 thus provides useful information for repurposing approved therapeutic agents as novel antiviral indications.

Naturally, drugs targeting viral proteins tend to be virus-specific. Drugs directed against cellular proteins or signaling pathways potentially have a much broader spectrum of antiviral activities, as the replication of different viruses often depends on similar cellular mechanisms. We next developed a computational approach (Fig 1F) to identify novel antiviral indications for existing drugs by incorporating drug-gene signatures from the Connectivity Map (CMap, build 02) [20] into the global virus-host interactome. The underlying hypothesis asserts that a drug would have a high potential for a specific antiviral indication if its related up-/down-regulated genes from CMap tended to be host genes that are essential for this virus replication (Fig 1E). Using *q* < 0.1 as a cutoff, we found 213 significant drug-virus pairs connecting 171 drugs and 29 viruses (S8 Table). Recently, He et al. experimentally identified 39 chlorcyclizine analogs with 50% maximal effective concentration (EC_50_) less than 100 μM for the treatment of hepatitis C virus (HCV) infection [57]. Herein, we computationally repurposed 11 potential drugs for anti-HCV infection with *q* < 0.1. Among 11 significant candidates, a drug homochlorcyclizine (7^th^-most significant prediction, *q* = 0.046) was previously reported to have the high anti-HCV activity with an EC_50_ value of 0.47 μM [57]. In addition, among the top 90 predicted drugs, three hits, including homochlorcyclizine (EC_50_ = 0.47 μM), clemizole (EC_50_ = 7.15 μM), and orphenadrine (EC_50_ = 10.5 μM) were validated (S8 Table), suggesting a higher enrichment (odds ratio = 3.3, *P* = 0.07; Fisher’s exact test) occurred in our computational approach, compared to the traditional experimental screens [57]. We next evaluated 2 case studies to discover new anti-HIV-1 and anti-Ebola indications for existing drugs.

### Identifying new anti-HIV-1 indications for existing drugs

Our bioinformatics analyses identified 16 drugs that have potential anti-HIV-1 indication (*q* < 0.1, S8 Table). Alsterpaullone, a small molecular cyclin-dependent kinase inhibitor, was significantly predicted to have an anti-HIV-1 indication (*q* = 0.011), validated by a previous study [58]. Lycorine, a toxic crystalline alkaloid, was predicted to have anti-HIV-1 activity, with the fourth-lowest *q* = 0.014 observed. A previous study has suggested that the amary-llidaceae alkaloid lycorine isolated from the bulbs of *Leucojum vernum* possesses anti-HIV-1 activity in MT4 cells with an IC_50_ value of 0.4 μg/mL [59]. Sanguinarine, a toxic quaternary ammonium salt, was predicted to have an anti-HIV-1 indication, with the fifth-lowest *q* = 0.019. Tan et al. found that sanguinarine nitrate shows moderate inhibitory activity, with an IC_50_ of 50–150 μg/mL against the HIV-1 reverse transcriptase [60]. Thus, among the top 5 predicted candidates, 4 agents have been validated in previous studies, indicating the possibility that other top candidates have anti-HIV efficacy as well. In addition, we systemically searched top 20 predicted agents for potential anti-HIV indications. S9 Table shows that 6 additional agents have demonstrated experimental anti-HIV activity data, including fursultiamine (*q* = 0.055), trichostatin A (*q* = 0.068), doxorubicin (*q* = 0.071), promethazine (*q* = 0.081), 8-azaguanine (17^th^ highest significance, *q* = 0.103), and staurosporine (20^th^ highest significance, *q* = 0.145), suggesting a 50% success rate in computational prediction for the top 20 candidates. Taken together, these data suggest potential application of our method in identifying anti-HIV-1 indications for existing drugs as well.

### Identifying new anti-Ebola virus indications for existing drugs

**Fig 8:**
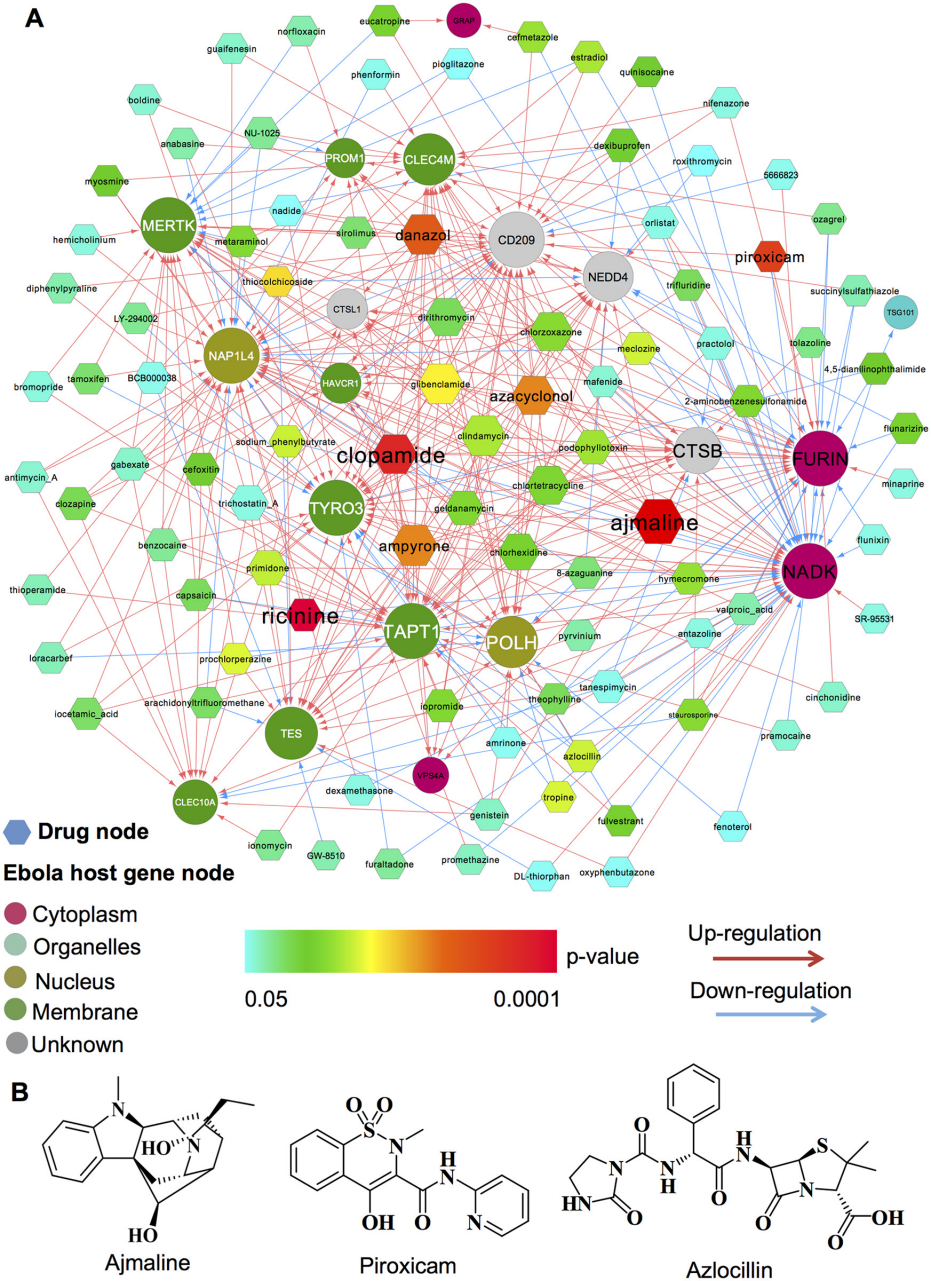
Novel drug-target interaction network for inhibiting anti-Ebola virus replication. (**A**) The newly discovered anti-Ebola virus drug-target interaction network, where nodes represent drugs (hexagons) or host genes (circles), and edges represent up-regulated (red lines) or down-regulated (blue lines) genes following drug treatment, as determined using the Connectivity Map data [20]. Target gene product nodes were colored based on their subcellular locations, and drug nodes were colored based on *P* values (Fisher’s exact test) calculated by our proposed computational approach (Fig 1E). (**B**) Chemical structures for three example drugs with significant *P* values. Detailed data is provided in S8 Table.

Infection by filoviruses such as the Ebola or Marburg viruses rapidly causes fatal hemorrhagic fever in humans, for which no approved small-molecule antiviral agents are available [4]. There is an urgent need to develop novel anti-Ebola virus agents, especially small molecule inhibitors. Herein, 7 agents were predicted to have potential anti-Ebola indications, with *q <* 0.1 (S8 Table). The top 5 agents identified were ajmaline (*q* = 0.002), ricinine (*q* = 0.008), clopamide (*q* = 0.016), piroxicam (*q* = 0.029), and danazol (*q* = 0.053). Fig 8 revealed that ajmaline up-regulates expression of several important Ebola-related genes, such as *MERTK*, *FURIN*, *TYRO3*, *FURIN*, and *CTSB* [61–63]. Recently, a bisbenzylisoquinoline alkaloid, tetrandrine, was found to inhibit entry of Ebola virus into host cells *in vitro* and preliminary studies in mice further confirmed the therapeutic efficacy against Ebola by inhibiting two pore calcium channel protein [64]. Moreover, ajmaline (≤ 20 μ M) exerted comparable pharmacological activity compared with tetrandrine (5–10 μ M) by inhibiting calcium channel protein activity [65]. Taken together, targeting *MERTK*, *CTSB*, *TYRO3*, and *FURIN* by alkaloid ajmaline may provide a novel therapeutic strategy against Ebola virus. Piroxicam, a non-steroidal anti-inflammatory drug, up-regulates Ebola-related genes: *FURIN* and *MERTK*. Azlocillin, an acylampicillin antibiotics with an extended spectrum of antibacterial activity up-regulates *NADK* and *POLH* expression and down-regulates *TAPT1* and *TYRO3* expression (Fig 8). Collectively, targeting *MERTK*, *CTSB*, *TYRO3*, and *FURIN* by existing agents (e.g. ajmaline) may provide potential strategies for Ebola virus prevention and therapy. Further study will be needed to provide experimental validations, which we hope will be prompted by the findings herein.

## Discussion

We here have demonstrated the use of a computable representation of genetic testing to effectively identify candidate antiviral targets and new antiviral indications for existing drugs. Our discovery pipeline represents a tight integration of gene-trap insertional mutagenesis testing and systems biology-based analysis. We total identified over 700 candidate host genes mediating the cytotoxic effects of 10 viruses, 1 bacterium, and 5 toxins (Figs 1D and 2). To further evaluate the quality of host genes identified by gene-trap, we performed several complementary systems biology-based analyses, including pathway-enrichment analysis, protein interaction network topological analysis, and protein evolution analysis. Fig 3 showed similar viral replication-related pathways, comparable connectivity distribution, and evolutionary features for the 712 trapped genes compared to three previous independent virus gene sets identified by RNAi, viORFs, and Co-IP+LC/MS. Recently, our group identified poliovirus receptor-like 3 (PVRL3) as a cellular factor necessary for *Clostridium difficile* TcdB-induced cytotoxicity using gene-trap insertional mutagenesis [22]. Furthermore, two genes discovered as being important in viral replication, *RAB9* [18] and *ADAM10* [66], have been experimentally validated by our group and others. In summary, we provided various complementary bioinformatics analyses to show the reliability of host genes identified by gene-trap insertional mutagenesis. However, several factors may potentially influence the data quality of candidate cellular genes identified by gene-trap insertional mutagenesis. For example, the use of different host species and tissues can confound and potentially invalidate some proteins in our network and bioinformatics analyses. Specifically, evolutionarily conserved genes involved in cellular replication are likely to be selected. Further studies will be needed to provide more experimental validation for candidate cellular genes identified by gene trapping, which we hope will be prompted by the findings herein.

Human viruses intrinsically depend on host cells during infection, and tumor viruses often cause cancer during replication. Herein, we examined viral perturbations in the host protein interaction network to test the hypothesis that genomic alterations and viruses may cause cancer through related mechanisms. For example, viral perturbations of the innate immunity protein interaction network revealed potential cancer etiologies (Fig 6). The proteins encoded by several cancer-related genes (*CTCF*, *RHOA*, *CDKN1B*, and *CUX1*) were also implicated in viral replication identified by gene-trap, consistent with a previous report that host interactome and transcriptome network perturbations caused by DNA tumor virus proteins impair Notch signaling and apoptosis pathways, a conserved process in cancer [45]. In addition, viral perturbations of the innate immunity protein interaction network also revealed novel insights into targeted cancer therapy, such as oncolytic reovirus-based cancer biotherapy [67]. For example, a gene *CTCF* was disrupted in a clonal cell lines resisting lytic reovirus infection (Fig 6). Recently, several cancer genome projects showed that *CTCF* mutations are significantly associated with breast cancer [48], head and neck cancer [49], and uterine cancer [50]. Previous studies showed that oncolytic reovirus infection induce tumor regression in several cancer types [51,67–69]. Collectively, the viral perturbations of the innate immunity protein interaction network presented in Fig 6 suggest potential mechanisms whereby oncolytic reovirus therapy may provide potential anticancer indications for multiple cancer types. However, there is also a confusing association with *CTCF* in relationship between viral replication and cancer. For instance, two recent studies indicated that *CTCF* expression level might also contribute to B-cell differentiation as well as Epstein-Barr virus latency type determination [70,71]. Many factors are associated with viral replication, while some viruses may exploit mutations or natural sequences of gene expression that may be cell-type specific. For example, reovirus may be inhibited in its replication by mutations that interfere with binding of *CTCF*, whereas the interaction with the DNA genome of Epstein-Barr virus directly, may lead to more complex interactions, dependent upon additional factors. Thus, further wet-lab experimental validation and deep computational analysis of biological consequences of *CTCF* involving in relationship between viral replication and cancer will be required before oncolytic reovirus therapy can be used in the clinic.

Although antiviral drug-discovery approaches have yielded notable successes in recent years, in many cases (such as with Ebola virus) no small molecular drugs are available to combat infection. However, systems biology-based antiviral drug repositioning enabling the identification of new antiviral indications for existing drugs will undoubtedly have a significant impact in antiviral drug discovery and expedite drug development. Here, we developed an integrated approach to identify new antiviral indications for existing drugs by incorporating drug-gene signatures into the global virus-host interactome (Fig 1E). We computationally identified numerous antiviral indications, several of which have already been validated in previous reports. For example, anti-HIV-1 indications have been already demonstrated for 3 most significantly predicted drugs. In addition, we further focused on novel drug indications for inhibiting Ebola virus in light of the Ebola outbreak in early 2014, for which no approved antiviral agents are available. We here computationally identified three small molecule drugs (e.g. ajmaline, piroxicam, and azlocillin) as novel drug candidates for anti-Ebola virus treatment, and the molecular mechanisms whereby these drugs may inhibit Ebola infection are provided in Fig 8. However, the CMap (v 2.0) used in this study only contained only approximately 7,000 expression profiles representing 1,309 compounds tested in 4 different cell lines. Recently, the Library of Integrated Cellular Signatures (LINCS) [72] has generated over one million genome-wide expression profiles representing more than 10,000 drugs tested in approximately 80 different cell lines. Our group is actively conducting computational analysis by utilizing this LINCS dataset. In summary, these data provide an integrated antiviral drug discovery pipeline by incorporating gene-trap and drug-gene signatures to successfully identify potential antiviral indications for existing drugs, although wet-lab experimental validation and clinical trials will be required before these drugs can be used in the clinic.

## Methods

### Cell culture, pathogens, and toxins

TZM-bl cells were obtained from the NIH AIDS Research and Reference Reagent Program (Germantown, MD). HepG2, Hep3B, L, MDCK, Sup-T1, and Vero E6 cells were obtained from the American Type Culture Collection (ATCC; Manassas, VA). Cowpox virus (Brighton strain), human rhinovirus 2 (HGP strain), human rhinovirus type 16 (11757 strain), and poliovirus (Chat strain) were obtained from the ATCC. *Herpes simplex* virus type 1 (KA Strain) was kindly provided by Dr. David Knipe (Harvard University). *Herpes simplex* virus type 2 (186 strain) was a gift from Dr. Patricia Spear (Northwestern University). Reovirus type 1 (Lang strain) was obtained from Bernard N. Fields. Ebola virus (Zaire species, 1976 Mayinga strain) and Marburg virus (1967 Voege strain) were studied in a BSL4 containment facility at the Centers for Disease Control in Atlanta, GA. The U3neoSV1 retrovirus shuttle vector [73] was obtained from H. Earl Ruley (Vanderbilt University) and was used as an insertional mutagen to prepare gene-trap libraries with parental, virus-sensitive cells, as described [18,74–76].

### Production of clonal gene-trap library cell lines resistant to lytic viral infection or toxin exposure

Methods describing the preparation of clonal gene-trap library cell lines resisting lytic infection using RIE-1 cells (reovirus), Sup-T1 (HIV-1), TZM-bl cells (human rhinovirus 2 and 16), and Vero E6 cells (cowpox, Ebola, *Herpes simplex* virus 1 and 2, Marburg, and poliovirus) were described previously [18,75–79]. Briefly, gene-trap libraries, each harboring approximately 10^4^ gene entrapment events, were expanded to 80–90% confluency until ~10^3^ daughter cells represented each clone. The indicated cell lines were infected with a low MOI (range = 0.0002–0.01), and infection proceeded until > 90% cytopathic effects were observed (3–7 days). The medium was changed every 2–3 days until surviving clones were visible, which were generally observed after 2–3 weeks in culture. Surviving clones were expanded in duplicate wells of separate 24-well plates, and resistance was confirmed in clones by re-infecting 1 of the duplicate wells at a 10-fold higher MOI than the original cell populations were exposed to. Resistant clones showing > 70% survival following re-infection were selected for expansion to identify trapped genes, using cells growing in the uninfected wells of 24-well plates.

Gene-trap library cells resisting cytolytic toxin exposure were prepared as follows. *Clostridium difficile*-TcdB toxin experiments were performed by first plating Caco-2 cells in 75 cm^2^ flasks and incubated with U3neoSV1 (multiplicity of infection, MOI = 0.1) at 37°C for 1 h in the presence of 4 μg/mL polybrene (Sigma), a cationic polymer used to increase the infection efficiency [80]. Next, gene-trapped Caco-2 cells were plated in 10-cm dishes and challenged with 15 nM native TcdB toxin for 4 h at 37°C, after which the medium was exchanged and the cells were left to recover for 96 h [22]. *Clostridium perfringens* ε toxin and *Staphylococcus aureus* α toxin experiments were performed after plating MDCK cells transduced with the gene-trap vector in nine 100-mm dishes (approximately 3.3 × 10^6^ cells per dish), in Leibovitz's L-15 medium. *Clostridium perfringens* ε toxin was added to a final concentration of 20 nM, and the treated cells were incubated at 37°C for 16 hours [74]. AZ-521 cells were infected for 1 h with *Helicobacter pylori* vacuolating toxin at an MOI of 0.1 in the presence of 4 μg/ml of polybrene. THP-1 cells were infected with *Francisella tularensis* at three different MOI values. Native ricin holotoxin was obtained commercially or purified from extracts of developing *Ricinus communis* seeds by standard procedures using a column of propionic acid-treated Sepharose 6LB, followed by specific elution of the cytotoxic lectin with 50 mM N-acetylgalactosamine. Recombinant ricin A chain variants (e.g., carrying C-terminal sulfation sites and glycosylation sequins) were prepared by expressing the ricin A chain cDNA in *Escherichia coli*. After incubating each cell type with indicated toxin or bacterium, resistant clones were expanded in separate wells of multi-well plates. The detailed protocols were described previously [18,75–79].

### Rescue and sequencing the U3neoSV1 shuttle vector from resistant clones

Genomic DNA from clonal, virus-resistant cell lines was extracted using the QIAamp DNA Blood Mini Kit (Qiagen, Inc., Valencia, CA). Shuttle vectors and genomic DNA fragments flanking the U3neoSV1 integration site were recovered by digesting genomic DNA with either BamH1 or EcoRI, self-ligating the resulting genomic DNA fragments, transforming *Escherichia coli*, and selecting for bacteria harboring carbenicillin-resistant plasmids, as described [75]. DNA sequences flanking the U3neoSV1 integration sites were sequenced using primers annealing to the U3neoSV1 shuttle vector.

### Sequence analysis

Genomic sequences obtained from shuttle clones were analyzed by the RepeatMasker (http://www.repeatmasker.org/cgi-bin/WEBRepeatMasker), followed by nucleotide-nucleotide BLAST searches against the National Center for Biotechnology Information (NCBI) database (http://www.ncbi.nlm.nih.gov). Virtually all genes that we identified matched murine and human sequences with probability scores (*P*) of <10^−10^ and <10^−20^, respectively. Detailed descriptions of this process were provided previously [18,22,74,80].

### Construction of a high-quality human protein interactome

We downloaded protein-protein interaction data from various publications and bioinformatics databases. Because the current publicly available human protein interaction databases are still incomplete, we constructed 5 different yet complementary human PINs: (i) a large-scale physical PIN, (ii) a three-dimensional structural PIN, (iii) a kinase-substrate interaction network (KSIN), (iv) a comprehensive innate immunity PIN, and (v) a large-scale computationally predicted PIN, based on our previous studies [25,26]. We implemented 2 data cleaning steps. First, we defined high-quality interactions as those that have been experimentally validated in human models through a well-defined experimental protocol. Interactions that did not satisfy this criterion were discarded. Second, we annotated all protein-coding genes using gene Entrez ID, chromosome location, and the official gene symbols from NCBI database (http://www.ncbi.nlm.nih.gov/), as described in detail previously [25,26].

### Construction of the drug-gene interactome

Drug-gene interactions (DGI) were acquired from the DrugBank database (v3.0) [81], the Therapeutic Target Database (TTD, v4.3.02) [55], and the PharmGKB database (December 30, 2014) [56]. Drugs were grouped using ATC classification system codes and annotated using Medical Subject Headings (MeSH) and Unified Medical Language System (UMLS) vocabularies (November 1, 2014) [82]. All genes were mapped and annotated using the gene Entrez ID and official gene symbols found in the NCBI database. All duplicated DGI pairs were removed. In total, we obtained 17,490 DGI pairs connecting 4,059 FDA approved or investigational drugs and 2,746 gene products.

### Categories of different disease gene sets

#### Cancer driver genes

A set of 384 genes that are significantly mutated in cancer was selected from several large-scale cancer genomic analysis projects [83–86].

#### Other cancer genes

Additional cancer genes were selected for bioinformatics analysis from the following resources. First, 560 experimentally validated cancer genes were downloaded on December 18, 2015 from the Cancer Gene Census [87] and denoted as CGC genes. We also collected 4,050 cancer genes assembled in a previous study [25], referred to here as the comprehensive catalogue of cancer genes, CCG set. Together, these resources provide overlapping and complementary candidate cancer genes.

#### Mendelian disease genes (MDGs)

A set of 2,714 MDGs was downloaded from the Online Mendelian Inheritance in Man (OMIM) database [88] in December 2012. The OMIM database contained 4,132 gene-disease association pairs connecting 2,716 disease genes in 3,294 Mendelian diseases or disorders (December 2012).

#### Orphan disease-causing mutant genes (ODMGs)

We collected 2,123 ODMGs from a previous study [89]. The United States Rare Disease Act of 2002 defines a disease as an orphan disease that affects fewer than 200,000 individuals in the United States, the equivalent of approximately 6.5 people per 10,000 [90].

#### Essential genes

Essential genes (2,719) were compiled from the OGEE database [24].

#### Cell cycle genes

Human host cell cycle genes (986 genes) regulating G0/1, S, and G2 phase transitions were collected from a previous study identified by a genome-wide RNAi screening [35].

#### Innate immune genes

Human innate immunity genes (971) playing a critical role in the innate immune response were collected from InnateDB [27].

### Computing selective pressure and evolutionary rates

We calculated *dN/dS* ratios [91] to examine selective pressures on genes. Initially, human-mouse orthologous genes were used to compute *dN* and *dS* substitution rates using human-mouse sequence data for 16,854 genes available in the Ensemble BioMart database (http://useast.ensembl.org/biomart/martview/). In addition, evolutionary rate ratios were determined, as described in a previous study [92]. Details of data and analyses were provided in our previous publication [25].

### Inferring protein evolutionary origins

The evolutionary origin of a protein refers to the approximate date that the protein originated and can be inferred from phylogenetic analysis. We used the protein origin data from ProteinHistorian [93]. Specially, the origin (age) of a protein was estimated by considering 3 factors: the species tree, the protein family database, and the ancestral family reconstruction algorithm. Furthermore, evolutionary distances were calculated by comparing human sequences with orthologous sequences from other animals, as described [92].

### Computational identification of new antiviral indications for existing drugs

We collected drug-gene signatures from the Connectivity Map (CMap, build 02) [20]. The CMap is comprised of over 7,000 gene expression profiles from human cultured cell lines treated with various small bioactive molecules (1,309 total) at different concentrations, covering 6,100 individual instances. The CMap thus provides a measure of the extent of differential expression for a given probe set. The amplitude (*a*) was defined as follows:

$$a\;=\;\frac{t-c}{(t+c)/2}$$

where t is the scaled and thresholded average difference value for the drug treatment group and c is the thresholded average difference value for the control group. Thus, a = 0 indicates no differential expression, a > 0 indicates increased expression (up-regulation) upon treatment, and a < 0 indicates decreased expression (down-regulation) upon treatment. For example, an amplitude of 0.67 represents a two-fold induction. Drug gene signatures with amplitudes of > 0.67 were defined as up-regulated drug-gene pairs, and amplitudes <—0.67 reflected down-regulated drug-gene pairs. To build a complete virus-host interactome, we combined the 712 host genes identified in our gene-trap study with the 2,449 host genes that were extracted from the literature based on experiments on 54 viruses using RNAi. Detailed data information was provided in S1 Table. After removing the duplicated data, we obtained ~2,600 host genes, which were then used to build the global virus-host interactome. We then mapped probe sets into the global virus-host interactome. In total, we compiled ~500,000 drug-gene pairs from the CMap connecting 1,309 drugs and 2,600 virus target genes.

For each drug-virus pair, we counted the number of host genes targeted by a given virus, those that are up- or down-regulated by drug treatments, as well as overlapping or mutually exclusive pairs (Fig 1E). Next, we calculated *P* values by Fisher’s exact test-corrected *P* values using Bonferroni’s multiple comparison test in R package for each drug-virus pair. We then used *q* < 0.1 as a cutoff to identify significant drug-virus pairs for antiviral drug repositioning.

### Network topology measurements

Network theory proposes that there are 2 important components of networks, namely nodes and edges. We studied virus-host bipartite networks, wherein nodes represented viruses and host cellular genes, and edges denoted interactions found by gene-trap. For PIN studies, nodes were comprised of proteins and edges were based on known physical interactions, protein structure evidence, and phosphorylation. We calculated connectivity (degree) values using Cytoscape v3.0.1. Hubs were defined as nodes ranked in the top 20% in the connectivity distribution, based on two previous studies [25,26].

### Functional enrichment analysis

We used ClueGO [94], a Cytoscape (v3.0.1) plug-in, and Ingenuity Pathway Analysis software (http://www.ingenuity.com/), for enrichment analysis of genes in the Reactome or canonical KEGG pathways. A hypergeometric test was performed to estimate statistical significances, and all *P* values were adjusted for multiple testing using Bonferroni’s correction (q).

### Statistical analysis and network visualization

All statistical tests were performed on the R platform (v3.01, http://www.r-project.org/). All network visualizations were prepared using Cytoscape v2.8.3 (http://www.cytoscape.org/).

## Supporting Information

S1 Fig Venn diagram showing the relationship between 712 host genes (trapped genes) identified by gene-trap insertional mutagenesis and (A) innate immunity genes and (B) human essential genes. (PDF)

S1 Table Global pathogen-host interaction network (toxin-host interactions and virus-host interactions) identified by genome-wide gene-trap insertional mutagenesis and previously reported RNAi screens. (XLSX)

S2 Table Top 20 most significantly enriched Reactome pathways for 712 host genes identified by gene-trap insertional mutagenesis. (PDF)

S3 Table Top 20 most significantly enriched Reactome pathways for 2,443 host genes identified in previously published RNAi screening studies. (PDF)

S4 Table Network topological (connectivity) analysis in five independent protein interaction networks. (PDF)

S5 Table List of virus-host gene interactions involved in cancer and innate immunity PIN. (XLSX)

S6 Table Top 20 most significantly enriched KEGG pathways for 691 druggable host genes identified in previous RNAi screens and gene-trap insertional mutagenesis studies. (PDF)

S7 Table List of existing drugs targeting 691 virus target genes identified by gene-trap studies and previously reported RNAi screens. (XLSX)

S8 Table Details of computationally predicted antiviral indications for existing drugs. (XLSX)

S9 Table Anti-HIV activities of top 20 predicted candidates based on the evidence from the literature. (PDF)
