# Supplementary material for: Systems Biology-Based Investigation of Cellular Antiviral Drug Targets Identified by Gene-Trap Insertional Mutagenesis
Source: PLoS Comput Biol. 2016 Sep 15;12(9):e1005074. doi: 10.1371/journal.pcbi.1005074 (PMC5025164; doi:10.1371/journal.pcbi.1005074)

S1 Fig. Venn diagram showing the relationship between 712 host genes (trapped genes) identified by gene-trap insertional mutagenesis and (**A**) innate immunity genes and (**B**) human essential genes.

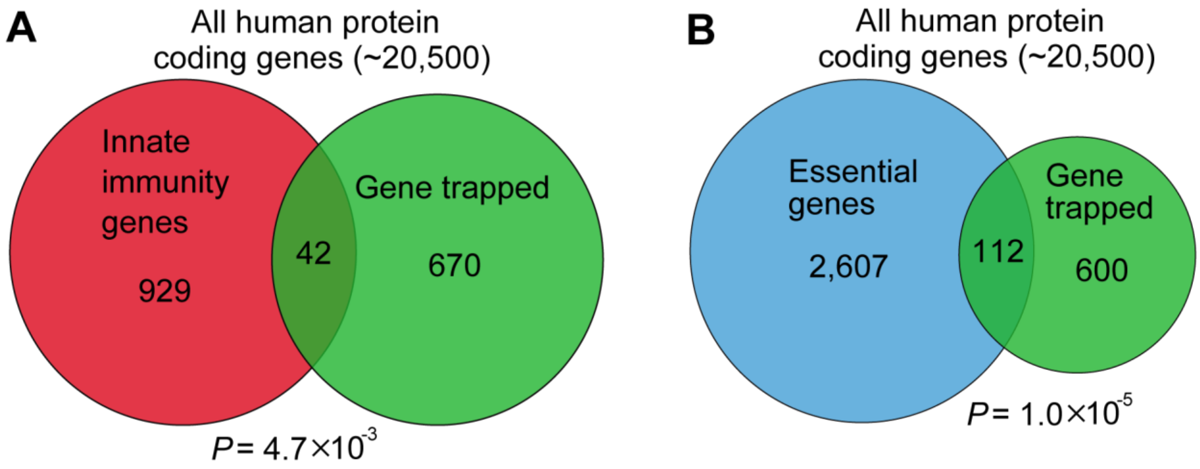

Supplement: S1 Fig — (PDF) [file pcbi.1005074.s001.pdf]
