# Supplementary material for: Systems Biology-Based Investigation of Cellular Antiviral Drug Targets Identified by Gene-Trap Insertional Mutagenesis
Source: PLoS Comput Biol. 2016 Sep 15;12(9):e1005074. doi: 10.1371/journal.pcbi.1005074 (PMC5025164; doi:10.1371/journal.pcbi.1005074)
Supplement: S2 Table — (PDF) [file pcbi.1005074.s003.pdf]

S2 Table. Top 20 significantly enriched Reactome pathways for 712 host genes identified by gene-trap insertional mutagenesis.

Pathway Source	GO Term	Term *P* value	Adjust *P* value (Bonferroni correction)
REACTOME_21.03.2014	Translation	2.10×10^{-10}	4.80×10^{-8}
REACTOME_21.03.2014	GTP hydrolysis and joining of the 60S ribosomal subunit	2.09×10^{-10}	4.82×10^{-8}
REACTOME_21.03.2014	Gene Expression	4.32×10^{-10}	9.86×10^{-8}
REACTOME_21.03.2014	Cap-dependent Translation Initiation	5.71×10^{-10}	1.30×10^{-7}
REACTOME_21.03.2014	Eukaryotic Translation Initiation	5.71×10^{-10}	1.30×10^{-7}
REACTOME_21.03.2014	L13a-mediated translational silencing of Ceruloplasmin expression	9.46×10^{-10}	2.13×10^{-7}
REACTOME_21.03.2014	Formation of a pool of free 40S subunits	9.43×10^{-10}	2.13×10^{-7}
REACTOME_21.03.2014	Peptide chain elongation	1.04×10^{-9}	2.34×10^{-7}
REACTOME_21.03.2014	SRP-dependent cotranslational protein targeting to membrane	1.36×10^{-9}	3.04×10^{-7}
REACTOME_21.03.2014	mRNA Splicing - Major Pathway	1.63×10^{-9}	3.61×10^{-7}
REACTOME_21.03.2014	mRNA Splicing	1.63×10^{-9}	3.61×10^{-7}
REACTOME_21.03.2014	Eukaryotic Translation Elongation	3.30×10^{-9}	7.29×10^{-7}
REACTOME_21.03.2014	Eukaryotic Translation Termination	4.79×10^{-9}	1.05×10^{-6}
REACTOME_21.03.2014	Viral mRNA Translation	4.79×10^{-9}	1.05×10^{-6}

REACTOME_21.03.2014	Nonsense Mediated Decay Independent of the Exon Junction Complex	1.00×10^{-8}	2.19×10^{-6}
REACTOME_21.03.2014	Influenza Viral RNA Transcription and Replication	3.18×10^{-8}	6.92×10^{-6}
REACTOME_21.03.2014	Influenza Infection	3.60×10^{-8}	7.81×10^{-6}
REACTOME_21.03.2014	Influenza Life Cycle	8.11×10^{-8}	1.75×10^{-5}
REACTOME_21.03.2014	Nonsense Mediated Decay Enhanced by the Exon Junction Complex	8.36×10^{-8}	1.80×10^{-5}
REACTOME_21.03.2014	Nonsense-Mediated Decay	8.36×10^{-8}	1.80×10^{-5}
