# Supplementary material for: Systems Biology-Based Investigation of Cellular Antiviral Drug Targets Identified by Gene-Trap Insertional Mutagenesis
Source: PLoS Comput Biol. 2016 Sep 15;12(9):e1005074. doi: 10.1371/journal.pcbi.1005074 (PMC5025164; doi:10.1371/journal.pcbi.1005074)
Supplement: S3 Table — (PDF) [file pcbi.1005074.s004.pdf]

S3 Table. Top 20 significantly enriched Reactome pathways for 2,443 host genes identified in previously published RNAi screening studies.

Pathway Source	GO Term	Term *P* value	Adjust *P* value (Bonferroni correction)
REACTOME_21.03.2014	HIV Infection	3.13×10^{-23}	1.57×10^{-20}
REACTOME_21.03.2014	HIV Life Cycle	3.38×10^{-15}	1.69×10^{-12}
REACTOME_21.03.2014	Host Interactions of HIV factors	5.20×10^{-14}	2.60×10^{-11}
REACTOME_21.03.2014	Disease	1.09×10^{-13}	5.42×10^{-11}
REACTOME_21.03.2014	Processing of Capped Intron-Containing Pre-mRNA	2.25×10^{-13}	1.12×10^{-10}
REACTOME_21.03.2014	Late Phase of HIV Life Cycle	1.33×10^{-12}	6.63×10^{-10}
REACTOME_21.03.2014	Immune System	1.10×10^{-11}	5.46×10^{-9}
REACTOME_21.03.2014	Antiviral mechanism by IFN-stimulated genes	7.60×10^{-11}	3.76×10^{-8}
REACTOME_21.03.2014	ISG15 antiviral mechanism	7.60×10^{-11}	3.76×10^{-8}
REACTOME_21.03.2014	Nuclear Envelope Breakdown	3.42×10^{-10}	1.69×10^{-7}
REACTOME_21.03.2014	mRNA Splicing - Minor Pathway	6.57×10^{-10}	3.24×10^{-7}
REACTOME_21.03.2014	Latent infection of Homo sapiens with Mycobacterium tuberculosis	1.08×10^{-9}	5.31×10^{-7}
REACTOME_21.03.2014	Phagosomal maturation (early endosomal stage)	1.08×10^{-9}	5.31×10^{-7}
REACTOME_21.03.2014	Nuclear Pore Complex (NPC) Disassembly	4.37×10^{-9}	2.15×10^{-6}
REACTOME_21.03.2014	Insulin receptor recycling	5.06×10^{-9}	2.48×10^{-6}
REACTOME_21.03.2014	Transcriptional Regulation of White Adipocyte Differentiation	5.44×10^{-9}	2.66×10^{-6}

REACTOME_21.03.2014	Activation of NF-kappaB in B Cells	7.31×10^{-9}	3.57×10^{-6}
REACTOME_21.03.2014	Regulation of mRNA Stability by Proteins that Bind AU-rich Elements	7.74×10^{-9}	3.77×10^{-6}
REACTOME_21.03.2014	Cytokine Signaling in Immune system	9.45×10^{-9}	4.59×10^{-6}
REACTOME_21.03.2014	Transport of Ribonucleoproteins into the Host Nucleus	1.55×10^{-8}	7.51×10^{-6}
