# Supplementary material for: Systems Biology-Based Investigation of Cellular Antiviral Drug Targets Identified by Gene-Trap Insertional Mutagenesis
Source: PLoS Comput Biol. 2016 Sep 15;12(9):e1005074. doi: 10.1371/journal.pcbi.1005074 (PMC5025164; doi:10.1371/journal.pcbi.1005074)
Supplement: S4 Table — (PDF) [file pcbi.1005074.s005.pdf]

S4 Table. Network topological (connectivity) analysis in five independent protein interaction networks.

Network	Number of Hub proteins	Number of non-hub proteins	Fisher's exact test
3DPIN	178	487	3.7×10^{-3}
INPIN	151	518	6.5×10^{-4}
KSIN	171	423	1.7×10^{-9}
PIN	760	1,506	4.3×10^{-66}
CPIN	846	1,601	3.6×10^{-76}

"Hubs" were defined as the nodes that are on the top 20% of the connectivity distribution in the network. 3DPIN: three-dimensional protein interaction network [1]; INPIN: innate immunity protein interaction network [2]; KSIN: kinase-substrate interaction network [3-6]; PIN: physical protein interaction network [2,7]; computationally predicted protein interaction network [8].

References

1. Meyer MJ, Das J, Wang X, Yu H (2013) INstruct: a database of high-quality 3D structurally resolved protein interactome networks. Bioinformatics 29: 1577-1579. doi: 10.1093/bioinformatics/btt181 PMID: 3673217.
2. Breuer K, Foroushani AK, Laird MR, Chen C, Sribnaia A, et al. (2013) InnateDB: systems biology of innate immunity and beyond--recent updates and continuing curation. Nucleic Acids Res 41: D1228-1233. doi: 10.1093/nar/gks1147 PMID: 3531080.
3. Dinkel H, Chica C, Via A, Gould CM, Jensen LJ, et al. (2011) Phospho.ELM: a database of phosphorylation sites--update 2011. Nucleic Acids Res 39: D261-267. doi: 10.1093/nar/gkq1104 PMID: 3013696.
4. Keshava Prasad TS, Goel R, Kandasamy K, Keerthikumar S, Kumar S, et al. (2009) Human Protein Reference Database--2009 update. Nucleic Acids Res 37: D767-772. doi: 10.1093/nar/gkn892 PMID: 2686490.
5. Newman RH, Hu J, Rho HS, Xie Z, Woodard C, et al. (2013) Construction of human activity-based phosphorylation networks. Mol Syst Biol 9: 655. doi: 10.1038/msb.2013.12.

6. Hornbeck PV, Kornhauser JM, Tkachev S, Zhang B, Skrzypek E, et al. (2012) PhosphoSitePlus: a comprehensive resource for investigating the structure and function of experimentally determined post-translational modifications in man and mouse. Nucleic Acids Res 40: D261-270. doi: 10.1093/nar/gkr1122 PMID: 3245126.
7. Cowley MJ, Pinese M, Kassahn KS, Waddell N, Pearson JV, et al. (2012) PINA v2.0: mining interactome modules. Nucleic Acids Res 40: D862-865. doi: 10.1093/nar/gkr967.
8. Lee I, Blom UM, Wang PI, Shim JE, Marcotte EM (2011) Prioritizing candidate disease genes by network-based boosting of genome-wide association data. Genome Res 21: 1109-1121. doi: 10.1101/gr.118992.110 PMID: 3129253.
