# Supplementary material for: Systems Biology-Based Investigation of Cellular Antiviral Drug Targets Identified by Gene-Trap Insertional Mutagenesis
Source: PLoS Comput Biol. 2016 Sep 15;12(9):e1005074. doi: 10.1371/journal.pcbi.1005074 (PMC5025164; doi:10.1371/journal.pcbi.1005074)
Supplement: S6 Table — (PDF) [file pcbi.1005074.s007.pdf]

S6 Table. Top 20 significantly enriched KEGG pathways for 615 potentially druggable host genes identified in previous RNAi screens and our gene-trap insertional mutagenesis studies.

Pathway Source	GO Term	Term *P* value	Adjust *P* value (Bonferroni correction)
KEGG_09.11.2015	Epstein-Barr virus infection	9.75×10^{-15}	2.01×10^{-12}
KEGG_09.11.2015	Chronic myeloid leukemia	2.28×10^{-12}	4.68×10^{-10}
KEGG_09.11.2015	Pathways in cancer	2.50×10^{-11}	5.10×10^{-9}
KEGG_09.11.2015	Proteasome	4.27×10^{-11}	8.67×10^{-9}
KEGG_09.11.2015	Prostate cancer	6.30×10^{-11}	1.27×10^{-8}
KEGG_09.11.2015	Pancreatic cancer	7.28×10^{-11}	1.46×10^{-8}
KEGG_09.11.2015	Prolactin signaling pathway	5.38×10^{-10}	1.08×10^{-7}
KEGG_09.11.2015	Osteoclast differentiation	5.50×10^{-10}	1.10×10^{-7}
KEGG_09.11.2015	Estrogen signaling pathway	9.18×10^{-10}	1.82×10^{-7}
KEGG_09.11.2015	Neurotrophin signaling pathway	1.06×10^{-9}	2.09×10^{-7}
KEGG_09.11.2015	ErbB signaling pathway	1.17×10^{-9}	2.30E-07
KEGG_09.11.2015	T cell receptor signaling pathway	3.02×10^{-9}	5.90×10^{-7}
KEGG_09.11.2015	Central carbon metabolism in cancer	4.53×10^{-9}	8.79×10^{-7}
KEGG_09.11.2015	Non-alcoholic fatty liver disease (NAFLD)	6.27×10^{-9}	1.21×10^{-6}
KEGG_09.11.2015	Proteoglycans in cancer	1.25×10^{-8}	2.40×10^{-6}
KEGG_09.11.2015	Glioma	1.52×10^{-8}	2.90×10^{-6}
KEGG_09.11.2015	PI3K-Akt signaling pathway	1.55×10^{-8}	2.94×10^{-6}

KEGG_09.11.2015	Influenza A	2.47×10^{-8}	4.67×10^{-6}
KEGG_09.11.2015	MAPK signaling pathway	4.52×10^{-8}	8.50×10^{-6}
KEGG_09.11.2015	Viral carcinogenesis	5.28×10^{-8}	9.88×10^{-6}
