# Supplementary material for: Systems Biology-Based Investigation of Cellular Antiviral Drug Targets Identified by Gene-Trap Insertional Mutagenesis
Source: PLoS Comput Biol. 2016 Sep 15;12(9):e1005074. doi: 10.1371/journal.pcbi.1005074 (PMC5025164; doi:10.1371/journal.pcbi.1005074)
Supplement: S9 Table — (PDF) [file pcbi.1005074.s010.pdf]

S9 Table. Anti-HIV activities of top 20 predicted candidates based on the evidence from literatures.

Predicted drug names	p-value	Adjusted p-value	Refs of Anti-HIV activity	Ranks
camptothecin	0.0000609	0.003715	[1,2]	Top1
sertaconazole	0.0000939	0.005728	NA	Top2
alsterpaullone	0.0001745	0.010642	[3]	Top3
lycorine	0.0002327	0.014195	[4]	Top4
sanguinarine	0.000319	0.01946	[5]	Top5
testosterone	0.0006199	0.037815	NA	Top6
amylocaine	0.000741	0.045201	NA	Top7
2,6-dimethylpiperidine	0.0007523	0.04589	NA	Top8
triprolidine	0.0009069	0.055322	NA	Top9
fursultiamine	0.0009652	0.058877	[6]	Top10
trichostatin A	0.0011253	0.068646	[7]	Top11
doxorubicin	0.0011878	0.071269	[8]	Top12
cobalt chloride	0.0012114	0.073893	NA	Top13
fulvestrant	0.0012775	0.07793	NA	Top14
promethazine	0.0013481	0.080886	[9,10]	Top15
viomycin	0.0013504	0.082375	NA	Top16
8-azaguanine	0.0016932	0.103283	[11]	Top17
0179445-0000	0.0019612	0.119631	NA	Top18
chlorphenesin	0.0022216	0.135518	NA	Top19
staurosporine	0.0023821	0.145307	[12,13]	Top20

References

1. Priel E, Showalter SD, Blair DG (1991) Inhibition of human immunodeficiency virus (HIV-1) replication in vitro by noncytotoxic doses of camptothecin, a topoisomerase I inhibitor. AIDS Res Hum Retroviruses 7: 65-72.

2. Li CJ, Wang C, Pardee AB (1994) Camptothecin inhibits Tat-mediated transactivation of type 1 human immunodeficiency virus. J Biol Chem 269: 7051-7054.

3. Guendel I, Agbottah ET, Kehn-Hall K, Kashanchi F (2010) Inhibition of human immunodeficiency virus type-1 by cdk inhibitors. AIDS Res Ther 7: 7. doi: 10.1186/1742-6405-7-7 PMID: 2852372.

4. Szlavik L, Gyuris A, Minarovits J, Forgo P, Molnar J, et al. (2004) Alkaloids from Leucojum vernum and antiretroviral activity of Amaryllidaceae alkaloids. Planta Med 70: 871-873. doi: 10.1055/s-2004-827239

5. Tan GT, Pezzuto JM, Kinghorn AD, Hughes SH (1991) Evaluation of natural products as inhibitors of human immunodeficiency virus type 1 (HIV-1) reverse transcriptase. J Nat Prod 54: 143-154.

6. Shoji S, Furuishi K, Misumi S, Miyazaki T, Kino M, et al. (1994) Thiamine disulfide as a potent inhibitor of human immunodeficiency virus (type-1) production. Biochem Biophys Res Commun 205: 967-975. doi: 10.1006/bbrc.1994.2760.

7. Kiernan RE, Vanhulle C, Schiltz L, Adam E, Xiao H, et al. (1999) HIV-1 tat transcriptional activity is regulated by acetylation. EMBO J 18: 6106-6118. doi: 10.1093/emboj/18.21.6106 PMID: 1171675.

8. Johansson S, Goldenberg DM, Griffiths GL, Wahren B, Hinkula J (2006) Elimination of HIV-1 infection by treatment with a doxorubicin-conjugated anti-envelope antibody. AIDS 20: 1911-1915. doi: 10.1097/01.aids.0000247111.58961.60.

9. Lu W, Achour A, Arlie M, Cao L, Andrieu JM (2001) Enhanced dendritic cell-driven proliferation and anti-HIV activity of CD8(+) T cells by a new phenothiazine derivative, aminoperazine. J Immunol 167: 2929-2935.

10. Aszalos A (2003) Phenothiazines in treatment of HIV infection (a review). Acta Microbiol Immunol Hung 50: 43-53. doi: 10.1556/AMicr.50.2003.1.5.

11. Wong RW, Balachandran A, Haaland M, Stoilov P, Cochrane A (2013) Characterization of novel inhibitors of HIV-1 replication that function via alteration of viral RNA processing and rev function. Nucleic Acids Res 41: 9471-9483. doi: 10.1093/nar/gkt727 PMID: 3814367.

12. Anizon F, Moreau P, Sancelme M, Voldoire A, Prudhomme M, et al. (1998) Syntheses,

biochemical and biological evaluation of staurosporine analogues from the microbial metabolite rebeccamycin. Bioorg Med Chem 6: 1597-1604.

13. Aranda-Anzaldo A, Viza D (1992) Human immunodeficiency virus type 1 productive infection in staurosporine-blocked quiescent cells. FEBS Lett 308: 170-174.
